# Evolutionary Origin of the Interferon–Immune Metabolic Axis: The Sterol–Vitamin D Link

**DOI:** 10.3389/fimmu.2017.00062

**Published:** 2017-02-09

**Authors:** Harry Newmark, Widad Dantoft, Peter Ghazal

**Affiliations:** ^1^Division of Infection and Pathway Medicine, School of Biomedical Sciences, University of Edinburgh, Edinburgh, UK

**Keywords:** sterol, metabolism, vitamin D receptor, cholesterol, xenobiotics, immunity

## Abstract

In vertebrate animals, the sterol metabolic network is emerging as a central player in immunity and inflammation. Upon infection, flux in the network is acutely moderated by the interferon (IFN) response through direct molecular and bi-directional communications. How sterol metabolism became linked to IFN control and for what purpose is not obvious. Here, we deliberate on the origins of these connections based on a systematic review of the literature. A narrative synthesis of publications that met eligibility criteria allowed us to trace an evolutionary path and functional connections between cholesterol metabolism and immunity. The synthesis supports an ancestral link between toxic levels of cholesterol-like products and the vitamin D receptor (VDR). VDR is an ancient nuclear hormone receptor that was originally involved in the recognition and detoxification of xenobiotic marine biotoxins exhibiting planar sterol ring scaffolds present in aquatic environments. Coadaptation of this receptor with the acquisition of sterol biosynthesis and IFNs in vertebrate animals set a stage for repurposing and linking a preexisting host-protection mechanism of harmful xenobiotics to become an important regulator in three key interlinked biological processes: bone development, immunity, and calcium homeostasis. We put forward the hypothesis that sterol metabolites, especially oxysterols, have acted as evolutionary drivers in immunity and may represent the first example of small-molecule metabolites linked to the adaptive coevolution and diversification of host metabolic and immune regulatory pathways.

## Introduction

Host-protection pathways against foreign harmful exogenous agents, inclusive of biotoxins and pathogens, exist in all branches of life. Pathways that allow the removal of biotoxins and metabolic by-products are considered to be distinct from those that neutralize and eliminate pathogens. For instance, it is understood that the P450 enzymes, which represent an ancient detoxification system, and interferon (IFN) pathways, that are central for immunity against infection in animals, are biologically unrelated. *However, could specific metabolic pathways and metabolites provide an interconnection?*

The substrates for P450 enzymes, while highly diverse, are lipophilic molecules often containing multiple planar ring structures. Notably, the most highly related P450s across the different kingdoms are involved in the metabolism of sterols (constituting multiple planar ring lipophilic molecules) and which further contribute an essential enzymatic role in the production of endogenous lipid metabolites, in particular as part of the sterol biosynthesis pathway (1–3). It has been debated whether the adaptation of P450 enzymes to the biosynthesis of sterols became firmly established in early eukaryotic (or late-stage prokaryotic) evolution with the arrival of atmospheric oxygen leading to the production of cholesterol in animals, ergo-sterol in fungi, and phyto-sterols in plants (4–7). The primary driver for sterol biosynthesis evolution was likely the selective advantage imparted by cholesterol toward modulation of membrane properties. However, too much cholesterol in membranes of cells, especially in the endoplasmic reticulum, the site of biosynthesis, can be highly toxic and accordingly sterol production, storage, and elimination is under stringent homeostatic regulation.

Sterols are not only required for membranes but also for the synthesis of steroid hormones, which regulate diverse physiological functions ranging from reproduction to stress and immunity. Outside the well-known functions of steroids, sterols, and in particular oxidized cholesterol and sterol metabolites, oxysterols, have been more recently found to have key roles in immunity (8, 9). Most importantly, the regulation of metabolic flux in cholesterol biosynthesis is directly linked to immune control through coupling to IFN signaling (10–13). Also see Robertson and Ghazal (14) for a review of our most current understanding of how IFN regulation is molecularly wired to sterol biosynthesis. We, therefore, posit that natural selection may have coadapted sterol metabolism and secondary metabolites as a link between functionally unrelated host-protection pathways in countering harmful chemical and biological agents. *This proposition evokes the question of whether there is evidence for an ancestral gene that supports a link between these distinct host-protection pathways?*

To address this question we sought to systematically review and provide a narrative synthesis of the literature based on investigating the ancestral connections between sterol metabolites, immunity, and xenobiotics. We find evidence supporting an evolutionary course for co-opting the ancestral, xenobiotic binding, vitamin D receptor (VDR) to adaptively recognize a specific non-typical oxysterol molecule, 1,25-dihydroxyvitamin D_3_, that in present day mammals governs prominent functions in calcium homeostasis, and immunity. It is important to clarify that vitamin D_3_ is a ring-opened version of 7-dehydrocholesterol and hence of the general class of sterols and steroids. For this reason and although vitamin D_3_ metabolites are not derived from cholesterol, we consider 25-hydroxyvitamin D_3_ (the inactive form found in serum) and 1,25-dihydroxyvitamin D_3_ (the active ligand to VDR) as non-typical oxysterols; as they are oxidized forms of the ring-opened cholesterol precursor 7-dehydrocholesterol.

On the basis of evidence presented, we further hypothesize that sterols and their oxidized metabolites have contributed as key evolutionary drivers for repurposing ancestral nuclear hormone receptors, in particular VDR, from protecting against harmful lipids to become important regulators of immunity.

## The Nuclear Hormone Receptor Family Connection

Central to the recognition of sterol-like molecules and activation of detoxification systems and immunity are the nuclear hormone receptors (15, 16). In particular, the subfamily known as NR1I that includes the pregnane X receptor (PXR), constitutive androstane receptor (CAR), and the VDR (15, 17). Although each have important individual functions in humans, these receptors act as important regulators of P450 enzymes and have strong genetic evidence suggesting they originated from a single ancestral nuclear receptor (18).

**Figure 1:**
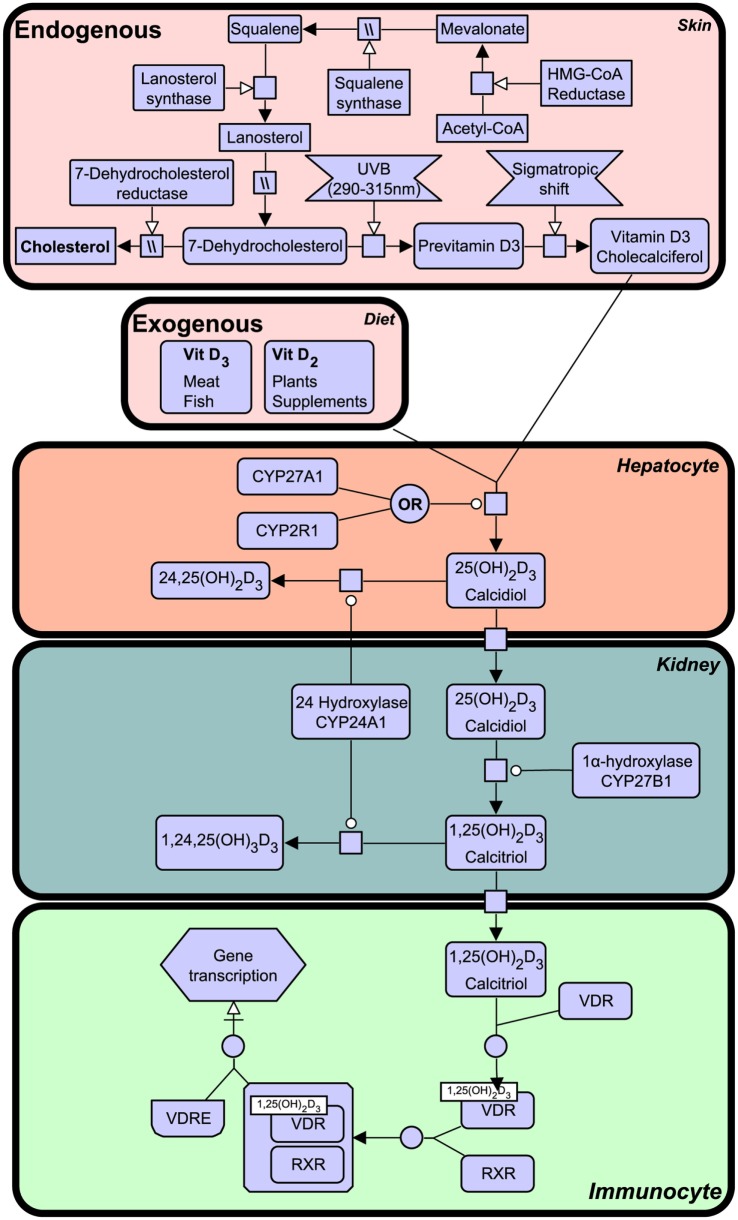
**The vitamin D synthesis pathway**. Vitamin D_3_ can be acquired both endogenously from cholesterol in the skin and exogenously through diet (vitamin D_2_ and vitamin D_3_). In the skin, 7-dehydrocholesterol, a cholesterol precursor, is converted to previtamin D_3_ upon ultraviolet B (UVB) exposure. Through a series of cytochrome P450 enzyme-mediated reactions, previtamin D_3_ is converted first into 25(OH)_2_D_3_ in liver hepatocytes and then activated in the kidney by 1α-hydroxylation, to form 1,25(OH)_2_D_3_. The degradation of 1,25(OH)_2_D_3_ and intermediate metabolites is mediated by negative feedback mechanisms (24). Figure created using the SBGN format on VANTED (25). See Table 1 for notation.

Notably, VDR that is activated by a specific ligand, 1,25-dihydroxyvitamin D_3_, generated from vitamin D that is derived from a precursor of cholesterol, 7-dehydrocholesterol from the sterol biosynthesis pathway and synthesis in humans begins in the skin upon exposure to ultraviolet B (UVB) light emitted from the sun. The vitamin D synthesis pathway is summarized in Figure 1 (for notation see Table 1), and involves the skin, liver, and kidneys (19). Interestingly, animals with fur and feathers are still able to synthesize vitamin D from sunlight despite UVB not reaching the skin (20). Here, vitamin D synthesis occurs through the sebaceous glands producing oily secretions (containing 7-dehydrocholesterol) that cover fur or feathers and ingested after grooming (21–23).

**Table 1:** **Systems biology graphical notation legend**.

Type	Symbol	Description
Entity pool nodes	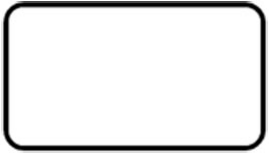	Macromolecule
	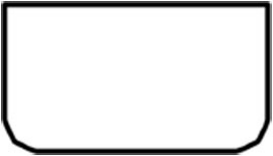	Nucleic acid feature
	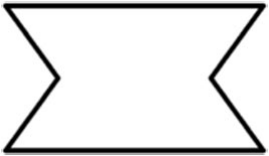	Perturbing agent
	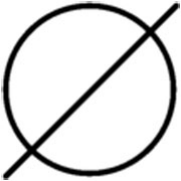	Source and sink

Container nodes	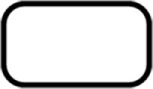	Compartment

Process nodes	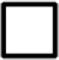	Process
	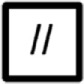	Omitted process
	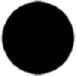	Association
	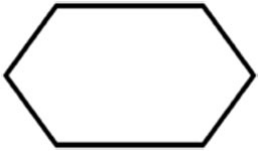	Phenotype

Connecting arcs	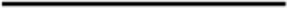	Consumption
	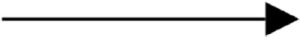	Production
	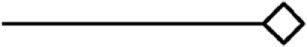	Modulation
	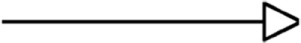	Stimulation
	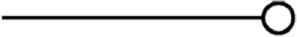	Catalysis
	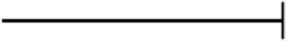	Inhibition
	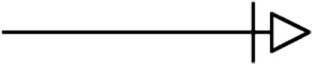	Necessary stimulation

Logical operators	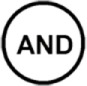	AND operator
	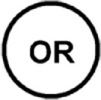	OR operator

Vitamin D deficiency can result in clinical disorders, the most notable being the characteristic bow-legged musculoskeletal manifestation known as rickets. Additional studies have also linked deficiency to cardiovascular disease, cancer, autoimmune conditions, and decreased antimicrobial protection (26–28). The VDR is known to heterodimerize with retinoid X receptor (RXR) and exerts biological effects as a ligand activated transcription factor by binding to specific vitamin D response element (VDRE) in gene promoters of over 200 genes (29). The active ligand of VDR is 1,25-dihydroxyvitamin D_3_ (1,25(OH)_2_D_3_), also known as calcitriol, although certain bile acids are also capable of inducing transactivation to a lesser degree (Figure 1). 1,25(OH)_2_D_3_ is the active form of vitamin D, produced by enzymatic hydroxylation of the circulating 25-hydroxyvitamin D_3_ by cytochrome P450 (CYP) enzyme 27B1 (see Figure 1).

While vitamin D and its receptor have been long regarded as mediators of calcium and phosphate homeostasis, VDR has additional roles in innate and acquired immunity and xenobiotics (30). Vitamin D-mediated calcium homeostasis has been around since the first terrestrial vertebrates, including amphibians, which have also been observed to suffer from calcium deficient ailments such as rickets (31). Before the calcium endocrine system, ancient VDR functioned as a xenobiotic receptor, mediating the degradation of marine biotoxins (32). It still retains this ability, and in humans VDR is important in detoxifying the toxic secondary bile acid lithocholic acid (LCA) in the colon by activating the CYP3A4 P450 enzyme (30).

Vitamin D is itself an ancient sterol–steroid, present in phytoplankton and zooplankton (33). VDR orthologs have likewise been observed in ancient vertebrates (34) and invertebrates (35). Accordingly, we next examine the origin and early evolutionary progression of VDR and its role in detoxification.

## Primordial Nuclear Hormone Receptor Family of the VDR and Biological Roles in Detoxification

**Figure 2:**
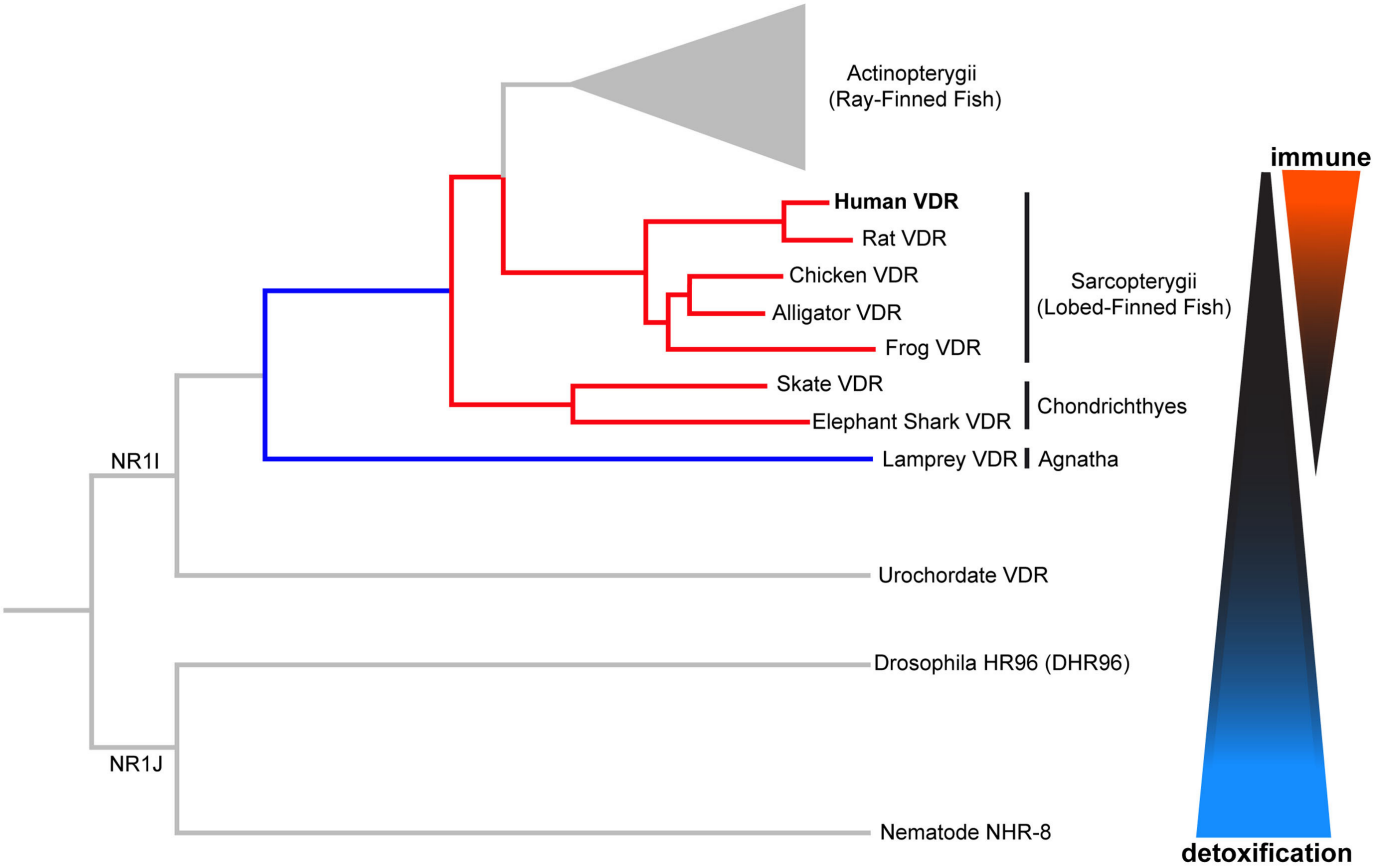
**Phylogenetic relationship of vertebrate vitamin D receptor (VDR) with invertebrate orthologs**. Both vertebrate and invertebrate VDR, belonging to the closely related NR1I and NR1J subgroups, respectively, regulate the expression of genes involved in xenobiotic metabolism (detoxification). Evolutionary change in the vertebrate VDR has resulted in the acquisition of new immunological functions. Distances between nodes are not to scale.

The VDR is descended from a group of xenobiotic nuclear hormone receptors known as NR1I, as shown in Figure 2. The NR1J nuclear receptor subfamily has important roles for xenobiotic detoxification in arthropods and nematodes (15, 16, 36). For instance, the related DHR96 receptor in *Drosophila melanogaster* can bind and detoxify a phenobarbital insult through a CYP transcriptional response (37), and NHR-8, in the *Caenorhabditis elegans* gut, senses colchicine and targets the activation of its cognate detoxification pathway (38). Many of these related nuclear receptors have also shown the ability to bind small lipophilic molecules such as cholesterol or steroid hormones (15, 16, 36). In this way, conservation of function can be seen throughout the evolution of these nuclear receptors. We will first discuss the ancestral xenobiotic role of VDR before considering how evolutionary pressures may have promoted the functional repurposing of this receptor with the acquisition of new roles including detoxification of endogenous compounds (e.g., vitamin D metabolites and bile acids), lipid regulation, and immunity.

**Figure 3:**
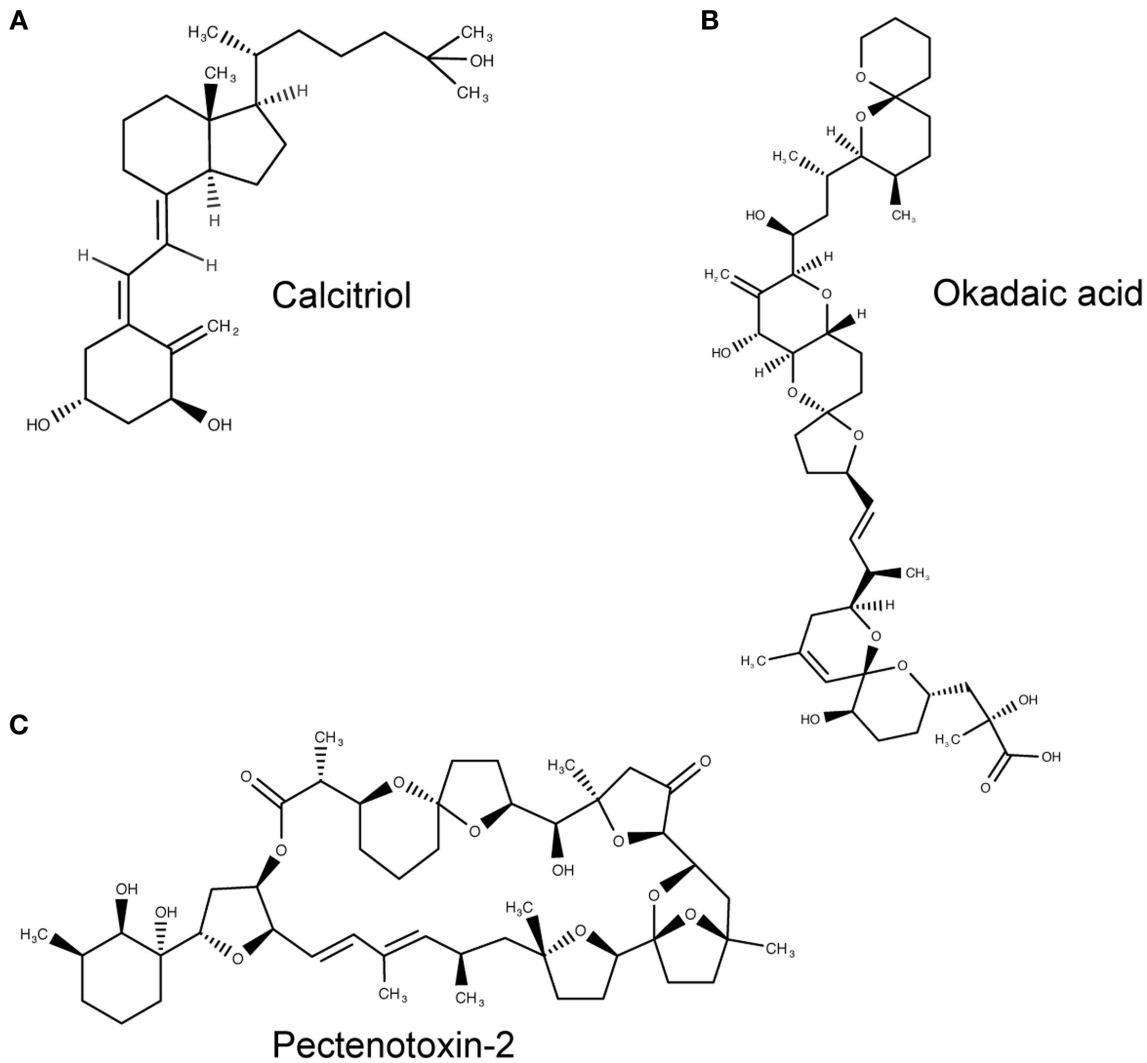
**Molecular structures of calcitriol, okadaic acid, and pectenotoxin-2**. **(A)** Molecular structure of 1,25(OH)_2_D_3_, otherwise known as calcitriol (PubChem CID = 5280453), a vitamin D receptor (VDR) agonist. **(B,C)** Atomic structure of two natural *Ciona intestinalis* CiVDR/PXRα analogs, okadaic acid (446512) **(B)** and pectenotoxin-2 (6437385) **(C)**, from left to right. Figures produced using MarvinSketch (http://www.chemaxon.com).

The tunicate, *Ciona intestinalis*, represents the closest extant invertebrate relative of vertebrates possessing an ancestral VDR gene (39). Fidler et al. (35) investigated the potential ligand-binding properties of this receptor, named as CiVDR/PXRα for its homology with both the VDR and PXR. Interestingly, vitamin D, or indeed any bile salts, were unable to produce any transactivation potential (40, 41). Despite speculation that the CiVDR/PXRα ortholog may be used for calcium homeostasis, there is evidence for closer functional similarity to PXRs current role in xenobiotics. This possibility is supported by the argument that the ocean is a plentiful source of calcium, making any need for homeostasis redundant. Present day PXR function in humans is to detoxify foreign toxic compounds by sensing and then activating the enzyme CYP3A4. There is good experimental evidence to support a functional role for the *C. intestinalis* CiVDR/PXRα ortholog to be ligand activated by microalgal biotoxins, including okadaic acid and pectenotoxin-2 (35) (Figure 3). Filter feeding tunicates like *C. intestinalis* accumulate these biotoxins through the large quantity of microalgae in their diet. As high concentrations of these chemicals are able to kill cells, CiVDR/PXRα’s ability to bind and detoxify them would be appropriate and consistent with PXRs current role in humans. Indeed, it has been shown that orthologous NR1Jβ receptors in mollusks likewise respond to xenobiotic insult from okadaic acid by activating detoxification pathways (42).

Similar to CiVDR/PXRα in *C. intestinalis*, NHR-8 and DHR96, members of the NR1J subfamily, in *C. elegans* and *D. melanogaster*, respectively, have also been shown to be essential for mediating xenobiotic resistance by promoting the expression of genes involved in metabolism of endo- and xenobiotics (37, 43, 44). Increased expression of genes involved in xenobiotic metabolism, together with resistance to xenobiotics, are frequently correlated with lifespan extension in *C. elegans, D. melanogaster*, and mice, suggesting detoxification of diet-acquired toxins is a host-protection mechanism against accumulation of specifically lipophilic toxins that negatively impact health during aging (The Green Theory of Aging) (16, 45). However, a recent study by Afschar et al. showed that DHR96 is indeed essential for mediating resistance to xenobiotics but not for increasing lifespan of insulin-mutant flies (44), indicating that xenobiotic resistance and longevity may not be causally connected. It has been suggested that the co-occurrence of xenobiotic resistance and lifespan extension may have co-evolved because lowered insulin/insulin-like growth factor signaling (IIS) can also signal the presence of pathogens (44). In line with this concept, in *C. elegans* and *D. melanogaster*, genes involved in xenobiotic metabolism have also been shown to be indirectly activated by toxic microbial by-products that directly cause dysfunction in cellular processes such as an altered metabolism, and decreased host translation and IIS (43, 46, 47).

## Evolutionary Repurposing of Primordial VDR from Exogenous to Endogenous Detoxification Pathways

Pharmacophore modeling of CiVDR/PXRα ligands revealed specific chemical scaffolds were required for receptor binding, comprising two hydrophobic features (in particular aromatic rings) and one hydrogen bond acceptor in a planar arrangement (35). The structure of activated vitamin D exhibits resemblance to this scaffold presenting a planar conformation with aromatic rings and hydrophobic features (48).

Further sequence identification by Ekins et al. (41) demonstrated a 67.6 and 17.1% similarity between the DNA binding domain (DBD) and the ligand-binding domain (LBD), respectively, between CiVDR/PXR and hVDR. Lower conservation of the LBD suggests evolutionary adaptive changes in ligand affinity. It is perhaps this promiscuous ligand-binding quality that allowed the ancestral VDR to function as a xenobiotic receptor, by binding and detoxifying new toxic chemicals on exposure. The similarities in structure between 1,25(OH)_2_D_3_ and exogenous marine biotoxins, alongside the genetic variability of VDRs’ LBD, allowed for the eventual binding and regulation of 1,25(OH)_2_D_3_ levels, a sterol-derived metabolite. As described below, observations in basal vertebrates provide insight toward understanding how this evolutionary pressure may have been applied.

The lamprey (*Petromyzon marinus*) is the most basal extant vertebrate, and therefore, provides valuable information regarding VDR functional evolution. The lamprey (lampVDR) has an 87 and 60% homology with hVDR DBD and LBD, respectively (18, 34). This differential increase in homology of the LBD in comparison with the tunicate CiVDR/PXR ancestral gene suggests VDRs’ ligand activated role may have changed. There have been three rounds of whole genome duplications (WGDs), represented as 1R, 2R, and 3R, since *C. intestinalis*. Humans diverged after the second round while Teleost fish were subjected to a third round and therefore, have an extra copy of the VDR gene (18). It is thought that after 1R, the combined VDR/PXR gene split giving rise to separate VDR and PXR genes with different but also overlapping functions. The lamprey was first to diverge after the 1R WGD event, as shown in Figure 2. While, a PXR homolog has yet to be identified in the lamprey, lampVDR has been shown to have high affinity binding and transactivation by 1,25(OH)_2_D_3_, which functionally activates CYP3A4, and possibly CYP24A1, in order to detoxify high levels of this sterol (34) and providing an opportunity for an extended regulatory role for the VDR in lipid metabolism. Although the lamprey lacks a calcium endocrine system, lampVDR may contribute to the regulation of other processes such as skin differentiation, as an observed increase in VDR presence in mucous glands and keratinized teeth has been reported (34, 49).

**Figure 4:**
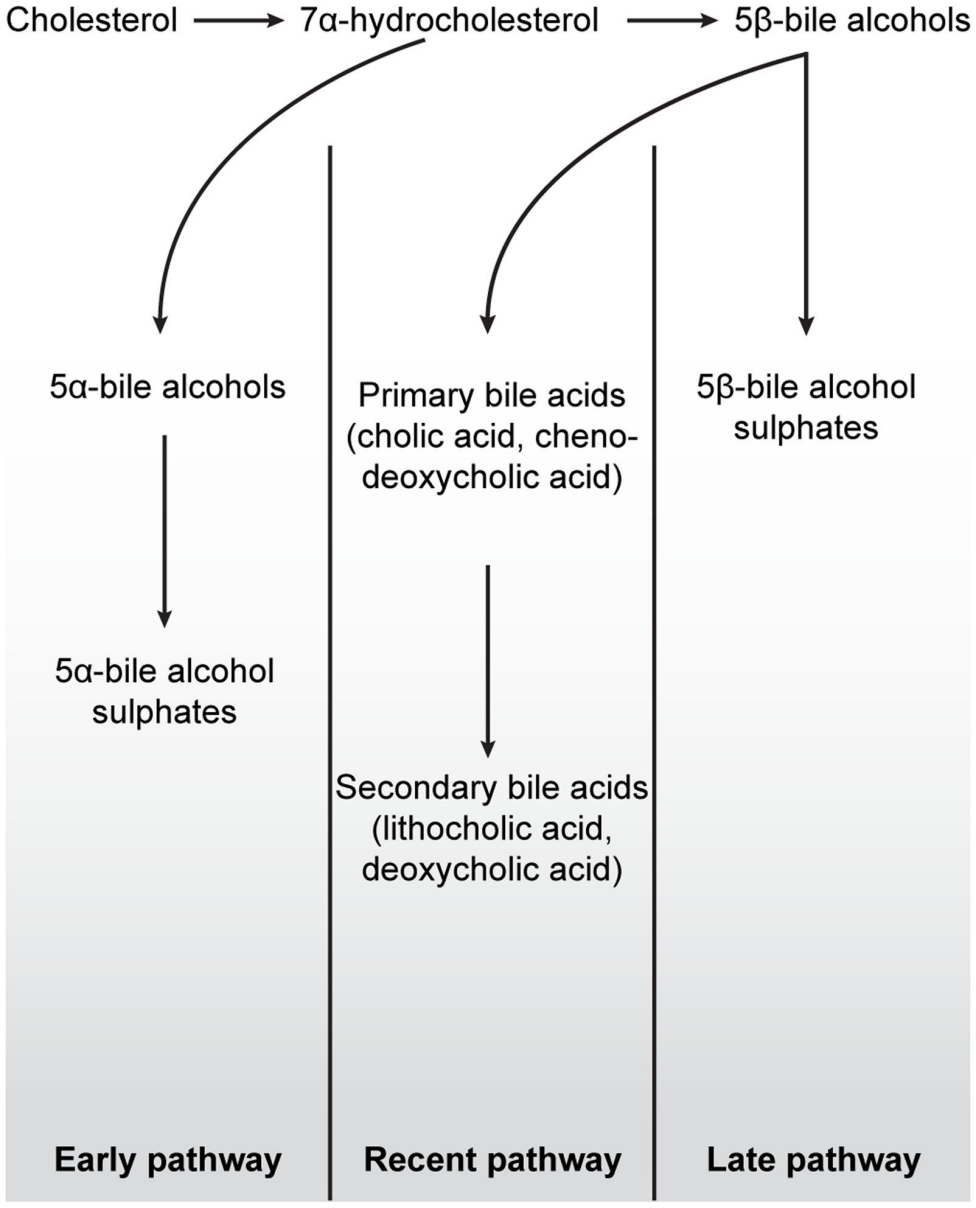
**Schematic overview of the three major bile salt pathways throughout vertebrate evolution**. All bile acids are derived from cholesterol, a 27-carbon molecule. In early fish, e.g., hagfish and sea lamprey, a 7α-hydrocholesterol is converted to 5α-bile alcohols, followed by conversion to 5α-bile alcohol sulfate. The production of bile salts in mammals, birds, cartilaginous fish, and in some teleost and amphibians is dependent on the conversion of 7α-hydrocholesterol into 5β-bile alcohol. In cartilaginous fish, mainly, but also in some teleost and amphibians, 5β-bile alcohol is further converted to 5β-bile alcohol sulfates (“Later pathway”). In mammals and birds, as depicted in the “Recent pathway,” bile salt production involves the conversion of 5β-bile alcohol to 24-carbon bile acids in the liver (cholic acid and chenodeoxycholic acid) by cytochrome P450-mediated oxidation. When these bile acids are secreted into the lumen of the intestine, cholic acid and chenodeoxycholic acid are converted, by colonic bacteria, to the secondary bile acids deoxycholic acid and lithocholic acid, respectively. While secondary bile acids in higher concentrations are potentially toxic to cells, they can, together with primary bile acids, be taken up into the blood stream and liver for re-secretion. In all pathways, the production of 7α-hydroxylation is the rate-limiting step in these reactions. Figure based on Ekins et al. (41).

Thus, in addition to the metabolism of exogenous xenobiotic compounds, VDR further acquired an ability to detoxify certain lipophilic endogenous molecules such as bile acids. Bile salt pathways are important vertebrate mechanisms by which cholesterol can be removed from the body. There are at least three evolutionary classified bile salt pathways, referred to as early, later, and recent pathways (41) (Figure 4). The lamprey uses the early fish pathway, while chondrichthyes, such as the Little skate, use the later pathway. In mammals, the “recent” pathway converts cholesterol to 24-carbon (C_24_) bile acids, which can subsequently be converted to toxic secondary bile acids in the intestine by resident microorganisms (50). Indeed, one of the major roles of the hVDR is its ability to detoxify the secondary bile acid LCA by transactivation of CYP3A4 (30). As LCA is a product of the most recent C_24_ bile acid pathway, basal vertebrates such as the lamprey, which employ the “early” or “later” fish pathways, are unable to bind this molecule with their VDR (51). In mammals, LCA affinity is almost certainly a more recent evolutionary adaption to changes in the gut microbiome resulting in the production of toxic secondary bile acids. This ability of VDR to subsequently transactivate the CYP3A4 gene in response to the binding of a toxic chemical traces an evolutionary path from VDRs’ ancestral xenobiotic function. In this regard, it is noteworthy that the NHR-8 and DAF-12 nuclear receptors in *C. elegans*, homologs of VDR and part of the NR1J subgroup, have convergently evolved to control and bind dafachronic acids, a bile acid look-alike, important in the life-cycle of this species (52, 53).

## Functional Diversification of VDR in Lipid Metabolism and Immunity

Vitamin D receptor’s ancestral roots clearly stem from its ability to recognize and regulate detoxification pathways for environmental toxic chemicals, as observed in both NR1I and NR1J subgroups. From this stemmed, its ability to bind and detoxify endogenous lipophilic molecules such as 1,25(OH)_2_D_3_ and bile acids using P450 enzymes. Sterol metabolites are important biological molecules requiring careful regulation for incorporation into cell membranes and steroid hormones. Accordingly, this opens a new opportunity for VDR to adopt a regulatory role through negative feedback mechanisms conferred by its ligand binding. The next section will discuss how sterol metabolites may have further coadapted VDR for driving the diversification of VDR functionality into lipid regulation, immunity, and bone prior to a role for VDR in calcium homeostasis (34).

**Figure 5:**
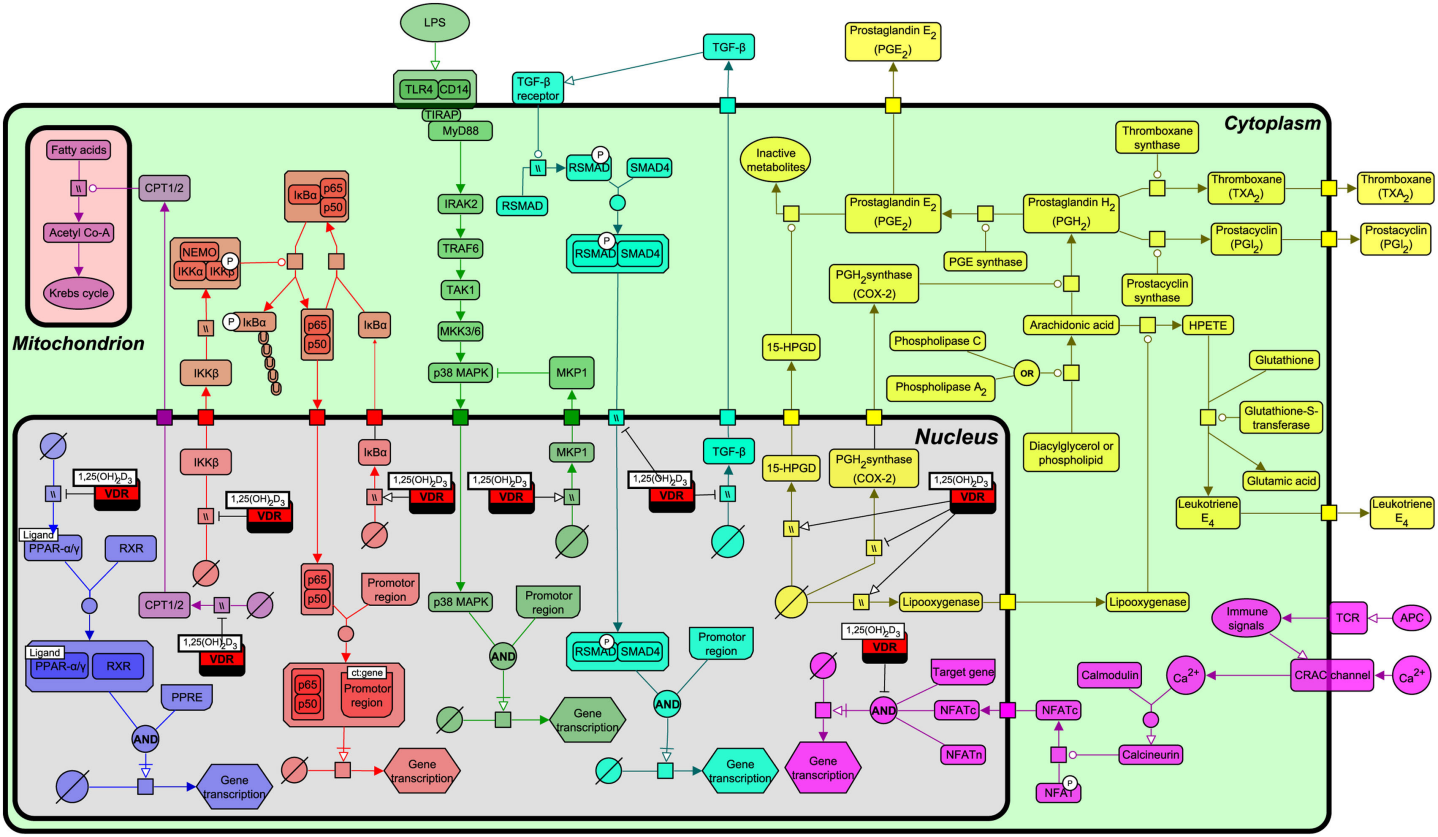
**Interactions of the vitamin D receptor (VDR) with PPAR-α, nuclear factor-kappa B (NF-κB), p38 mitogen-activated protein kinase (MAPK), transforming growth factor-beta (TGF)-β, β-oxidation, eicosanoid production, and nuclear factor of activated T-cells (NFAT) pathways**. From left to right, the pathways represented are PPAR-α/γ (blue), β-oxidation (light purple), NF-κB (red), p38 MAPK (green), TGF-β (turquoise), NFAT (dark purple), and eicosanoid synthesis (yellow). Figure created using the SBGN format on VANTED with edges representing high and low confidence interactions (25). See Table 1 for notation.

As described above, VDR origin is based on recognizing and regulating the levels of lipophilic exogenous and endogenous molecules through the transcriptional regulation of P450 enzymes. To more fully understand the functional relationship by which VDR interacts with various lipid metabolism and immune signaling pathways, we constructed a pathway biology diagram (Figure 5, for notation see Table 1) from the research synthesis of literature mapping all known interactions. Figure 5 shows that VDR is deeply embedded in a network of signaling pathways including PPAR-α and PPAR-γ, nuclear factor-kappa B (NF-κB), p38 mitogen-activated protein kinase (MAPK), transforming growth factor-beta (TGF-β), and eicosanoid synthesis. Of note, all of these pathways have instrumental roles in immunity, bone regulation, cell proliferation, and lipid metabolism.

A range of biological functions associated with VDR and targeted signaling pathways are shown in Table 2. Notably, most of these pathways not only regulate lipid metabolism, but have direct roles in immunity and bone homeostasis too. All these processes are interconnected. Lipids have a fundamental role in the immune system and directly affect immune cell function. They alter membrane fluidity, lipid peroxidation, gene expression, and eicosanoid production (54). Bone and immunity have a very close relationship, with bone marrow being a “high fat” primary hematopoietic tissue, controlling the production of B cells and other important innate and adaptive immune responses (55). Sterol biosynthesis is at the heart of this control network, using sterol-based interactions with the VDR to effectively regulate lipid metabolism and its associated features within bone and immunity. These pathways provide a link between the seemingly unconnected and multiple divergences of the VDR before additional more specific gene targets became evolutionary fixed and conserved, such as calcium homeostasis. In addition, it was also found that VDR has the ability to directly regulate fatty acid beta-oxidation by interacting with the enzymes hexokinase, CBT1, and CBT2, possibly leading to a role for energy metabolism in adipose tissue (56, 57).

**Table 2:** **The function of various signaling pathways and their corresponding interaction with the vitamin D receptor (VDR)**.

Pathway	Functions	Possible VDR interactions	Reference
PPAR-α PPAR-γ	Fatty acid metabolism Energy homeostasis Immune function Bone regulation	Protein–protein interactions between the ligand-binding domains of VDR and PPAR-α	(58) (59) (60) (61)

Nuclear factor-kappa B	Immune response Inflammation Cell cycle Bone regulation	VDR sequesters IKKβ, preventing NF-κB activation VDR modulates IκBα function, thereby controlling the translocation of NF-κB proteins	(62) (63) (64) (65)

P38 mitogen-activated protein kinase (MAPK)	Inflammation Skeletal muscle differentiation	VDR upregulates MAPK phosphatase-1 causing inactivation of p38 MAPK	(66) (67)

Transforming growth factor-beta	Cell growth and differentiation Inflammatory and immunological processes	Reduces TGF-β expression Inhibition of phosphorylated receptor-regulated SMAD Upregulation of SMAD6 Inhibition of SMAD2/3 nuclear translocation	(68) (69) (70) (71)

Eicosanoids	Inflammation Immune function Tissue growth Blood pressure	Inhibits cyclooxygenase-2 Stimulates 15-hydroxy-prostaglandin dehydrogenase Regulates expression of 5-lipoxygenase	(72) (73)

Nuclear factor of activated T-cells	Immune function Cell cycle Cytokine signaling	VDR/retinoid X receptor complex interacts with target gene and prevents NFAT binding.	(74)

It is worth noting that all these interactions are compartmentalized in terms of tissue specific, time dependent, and multifactorial control levels. This view is consistent with the possibility that cholesterol, as a toxic molecule and precursor sterols to vitamin D, repurposed VDRs’ ancestral function to provide a wider regulation over lipid metabolism and immune pathways.

Vitamin D receptor has long been known to promote immune tolerance in the acquired immune system while providing protective innate mechanisms against pathogen infection. The acquired immune functions of the VDR are complicated, involving the regulation of multifaceted signaling pathways such as PPAR-γ and NF-κB. The net outcome of these cross-regulatory responses results in attenuation of the immune adaptive response, involving stimulation of interleukin (IL)-10 and downregulation of IL-12 (75). The most notable case for innate immunity is the VDR function to induce expression of the cathelicidin antimicrobial peptide (CAMP) gene, an important host defense protein. Gombart et al. (76) demonstrated that exaptation of an AluSx short interspersed element provided a perfect VDRE in the *Camp* promoter. Moreover, VDR is upregulated during infection in a toll-like receptor 2/1 (TLR2/1)-dependent manner (77–79). This shows a direct connection to innate immunity and is where the historic treatment of tuberculosis with cod liver oil (high in vitamin D) may have a possible molecular explanation (80). Unsurprisingly, VDR-mediated innate immune responses have become targets of pathogen evasion techniques (81).

## The Connection with IFN Signaling

Interferons are a group of signaling proteins required for antiviral defense. Released by virally infected cells or leukocytes, they mediate a variety of innate and adaptive immunological responses by upregulating over 300 interferon-stimulated genes (ISGs) (82). IFNs can be classified into three types depending upon the receptor to which they bind and the signal transduction pathways they activate (83). Type I IFNs are split into multiple subtypes including -α, -β, -ω, -ε, -τ, -δ, and -κ, each with independent and redundant functions. Type II IFNs are conserved to just higher mammals and have only one member, IFNγ. Lastly, type III, containing IFN-λ genes (IL-29, IL-28A, and IL-28B), have similar biological properties as type I, but their genetic sequence contains non-coding intron sequences (84–86). The IFN system has displayed remarkable conservation throughout vertebrate evolution, demonstrating its importance for immunological defense (87). It is also inherently linked to cholesterol metabolism, as described elsewhere (8–10, 14).

Interferons appeared to have originated soon after the evolution of vertebrates as IFN homologous genes and their transcription factors have not yet been observed in primitive chordates such as the tunicate and sea urchin (87) or even closely related basal vertebrates such as the jawless fish, lamprey (88). We briefly discuss the evolution of type I and III IFN genes, as their ancestral homologs have coexisted with the evolving metabolic interactions between VDR, cholesterol, and immunity.

Interferon genes are present in many different varieties of fish, including the teleost clade of ray-finned fish that diverged from our evolutionary line 450 million years ago (87). Fish can possess singular or multiple IFN genes depending on the species and it is likely that vertebrate groups have independently evolved a vast array of structurally similar IFN molecules that perform different host protective functions (83, 89). Thus, the expanding role of IFNs coincided with the evolutionary changing roles of sterols and VDR.

Interferons act through the Janus kinase/signal transducer and activator of transcription (JAK/STAT) signal transduction pathways inducing and suppressing hundreds of genes. In mammals, IFNs are activated through a variety of pathogen pattern recognition systems, notably downstream of toll-like receptor (TLR) activation and by stimulator of interferon genes (STING)-activation that are now known to also target the sterol biosynthesis pathway. IFN signaling *via* STAT1 and IRF1 induces a cholesterol hydroxylase gene, CH25H and its cognate metabolite 25-hydroxycholesterol as well as a microRNA (miR342-5p) that dramatically suppresses the flux in the sterol biosynthesis pathway. A change in the flux of sterol biosynthesis in turn activates STING that further re-enforces the IFN response (14). It is worth mentioning that VDR can interact with STAT1 and curtail the nuclear translocation of ISGF3 and which may contribute to VDRs inherent immunosuppressive attributes (90). Furthermore, as mentioned above, numerous studies have reported on the importance of both IFN-expression and activation of the vitamin D-pathway on the expression of downstream effector molecules [e.g., antimicrobial peptides (AMPs)] that subsequently influence infection and inflammation (78, 91–99). Increased expression of type II IFN (IFNγ) have been correlated with macrophage activation, macrophage-dependent AMP gene expression, as well as with controlled growth of pathogenic intracellular microbes and better disease outcome (96, 97, 99). Fabri et al. reported that IFNγ, released by T cells induce in a vitamin D-pathway-dependent in human macrophages, autophagy, phagosomal maturation, and antimicrobial activity against *Mycobacterium tuberculosis* (97).

The role of IFN responses and the vitamin D-pathway has also been investigated in human leprosy. Teles et al. have revealed an inverse correlation between IFNβ, IL-10, and IFNγ, where IFNβ, in an IL-10-pathway-dependent manner, inhibited the IFNγ-induced and vitamin D-dependent, AMP response in disseminated and progressive lepromatous lesions (99). By contrast, IFNγ-specific genes were enriched in self-healing tuberculoid lesions. Both studies underscore the importance of adequate amounts of vitamin D in human populations for sustaining both innate and acquired immunity against infection. The close connection between IFN and the vitamin D-pathway have also been reported in experimental autoimmune encephalitis (EAE), a model for multiple sclerosis (MS) (100, 101), and diabetes (102), two non-infectious disorders characterized by excessive and uncontrolled inflammation and macrophage foam cell formation (characterized by accumulation of esterified cholesterol) (103, 104). Early studies have shown that IFNγ plays a crucial role in the induction of 1,25(OH)_2_D_3_ (105, 106), the active vitamin D metabolite that bind to VDR, in initializing VDR dimerization with RXR and, in VDR–RXR activation of VDRE-containing target genes (107–109). Adams et al. showed that IFNγ induces production of 1,25(OH)_2_D_3_ in macrophages and that the effect was abolished by addition of anti-IFNγ to the culture medium (110). The tissue availability of 1,25(OH)_2_D_3_ in immune cells is dependent on the expression of the activating enzyme 1α-hydroxylase (*Cyp27b1*) and its catabolic counterpart 25-OHD_3_-24-hydroxylase (*Cyp24a1*) (111). In addition to TLR signaling, expression of 1α-hydroxylase can be induced by IFNγ stimulation (112–114). Activation by IFNγ stimulation require, however, the cells to be differentiated, as IFNγ stimulation of undifferentiated monocyte THP1 cells failed to induce *Cyp27b1* expression (1α-hydroxylase) in the absence of a second stimulus [lipopolysaccharide (LPS)] (112). On the other hand, *Cyp24a1* (25-OHD_3_-24-hydroxylase) expression is induced by the type 2 T helper cell cytokine IL-4, in toll-like receptor 2/1 ligand-activated monocytes, but not by IFNγ (114). Stoffel et al. revealed that, in cultured monocytes, synergistic induction of *Cyp27b1* gene expression by IFNγ and LPS, required, not only activation of the JAK/STAT pathway and NF-κB binding but also binding of phosphorylated C/EBPβ (by the p38 MAPK pathway) (113).

In addition to regulation of VDR through the transcriptional regulation of *Cyp27b1, VDR* gene expression was shown to be dependent on the *IFNG* gene (101). *Ifng* knockout (GKO) mice exhibited very low *Vdr* gene expression in the central nervous system. Correlating with the low *Vdr* expression, GKO mice also demonstrated an increased pathogenic T cells burden as well as a more severe EAE phenotype, suggesting that the aggressive autoimmune CD4^+^ T cell phenotype may be a consequence of inadequate *Vdr* gene expression (101). Furthermore, treatment of TLR2/1L-activated monocytes with IFNγ has been shown to not only stimulate *Cyp27b1* expression but also *Vdr* gene expression (114). Collectively, these studies suggest that IFN-regulation of VDR activity is complex and regulated indirectly *via* regulation of 1α-hydroxylase, and possibly directly at the gene level by IFNγ-induced STAT binding.

## Present Day IFN-Sterol Metabolic Link

**Figure 6:**
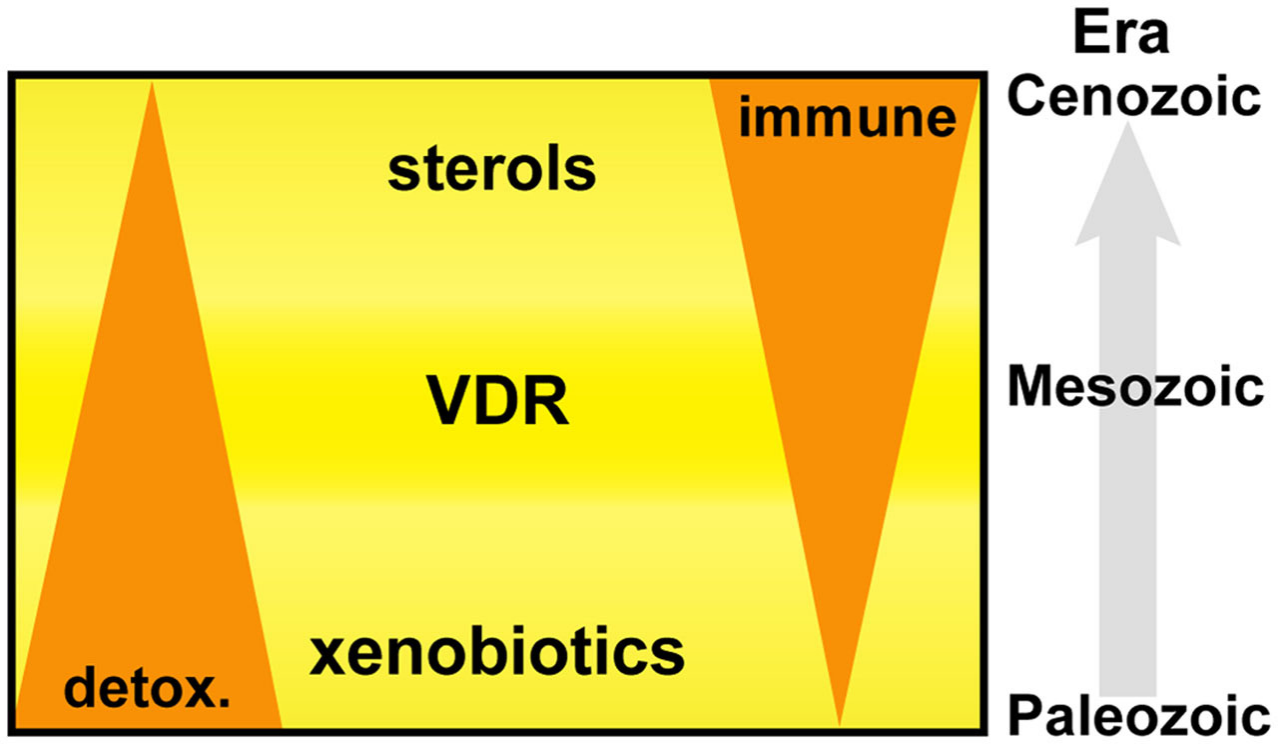
**Vitamin D receptor (VDR) is an ancient nuclear hormone receptor that was originally involved in the recognition and detoxification of xenobiotic marine biotoxins exhibiting sterol ring scaffolds present in aquatic environments**. Coadaptation of this receptor with the acquisition of sterol biosynthesis and interferons in vertebrate animals set a stage for repurposing and linking a preexisting host-protection mechanism of harmful xenobiotics to become an important regulator in immunity.

Primitive NR1I and NR1J receptors have inherent affinities for lipophilic molecules, making them useful xenobiotic sensors in vertebrate and invertebrate organisms. Figure 6 depicts the adaption of this receptor from the detoxification of endogenous compounds including bile acids and vitamin D in vertebrate organisms to acquiring new biological roles associated with the increasing importance of sterols and especially oxysterols for immune cell function. From this foundation, VDR continued to develop further direct and indirect interactions with lipid metabolism pathways, placing VDR in an opportunistic position to influence the development and regulation of other important biological systems including, but not limited to, immunity, bone development, and cell proliferation. The immune roles have been further consolidated with the acquisition of fixed mutations, such as the VDR regulated production of innate AMPs.

In conclusion, our findings support an evolutionary basis to the IFN–sterol–immune metabolic link, arising from the xenobiotic origins of a nuclear hormone receptor, VDR (Figure 6). Xenobiotics and immunity are considered separate pathways facilitating the removal of toxic molecules and pathogens, respectively. However, our narrative synthesis suggests that sterol metabolites acted as an evolutionary driver integrating a complex metabolic network under bi-directional IFN and VDR control. We believe this is likely to be a more general evolutionary mechanism for other nuclear hormone receptor functions in immunity such as glucocorticoid receptor, liver X receptor (LXR), and farnesoid X receptor (FXR) and possibly for other distinct immune directed metabolic pathways and associated ligand activated receptor systems.

## Concluding Remarks on the *General* Hypothesis of Microbial–Host Small-Molecule Metabolites as Functional and Evolutionary Drivers for Recognizing and Defending Against Non-Self

The question of whether a functional–evolutionary link between planar sterol-like molecules, immunity, and detoxification is specifically unique to VDR would implicate a limited selective role rather than one pertaining to a more central evolutionary principle with broader biological significance. Notably, in this context other evolutionary related subfamily 1 nuclear receptors, including LXR, FXR, PXR, CAR, retinoic-acid receptor (RAR), and RAR-related orphan receptor (32), similarly exhibit, at a number of levels, cross talk between mevalonate-sterol metabolism and immunity, as well as in xenobiotic detoxification [for examples see Ref. (115–118)]. Interestingly, RXRs partner with many of these receptors and which recruits corepressor or coactivator molecules to regulate transcriptional responses. Homologs and orthologs of this highly conserved nuclear receptor have been identified in marine and terrestrial invertebrates (119–123), so it is likely that RXR coevolved with VDR and the many other nuclear receptors it is associated with. RAR and RXR are activated by the vitamin A derived ligand, retinoic acid and its 9-cis conformer, respectively. While this lipophilic molecule is not a steroid-derived molecule, the heterodimerization of RXR provides an important but insufficient role of vitamin A metabolites in integrating with these receptor systems and sterol metabolism (124–127).

Of the related subfamily 1 nuclear receptors, strong conservation across vertebrate species can be found for the LXRs, with approximately 75% sequence identity in the LBD between human and non-mammalian LXRs (128). Consistent with the high degree of sequence conservation, ligand specificities between mammalian and non-mammalian vertebrates homologs, as well as between vertebrate and non-vertebrate LXR orthologs, are very similar (128). While vertebrate LXR agonists do not activate *Ciona* LXR, it is activated by a number of oxysterols as well as some pregnane and androstane steroids. In insects on the other hand, the ecdysone receptor (EcR) has been identified as the ortholog for both LXR and FXR combined (120). Together with its interaction partner, ultraspiracle (ortholog of RXR), EcR play an essential role in insect development and reproduction, as well as in basic metabolism and immunity (129, 130). In mammals, FXRs, with their high expression in liver, adrenal glands, intestine, and kidney, are activated by farnysol and its metabolites, part of the mevalonate–sterol biosynthesis pathway (131) and by primary bile acids such as chenodeoxycholic acid (132–134). Together with PXRs and VDR, FXR serves as one of the major transcriptional regulators of bile salt synthesis, partly by regulating the expression of CYP7A1 and CYP8B1 (135). Bile salts have to date not been detected in invertebrates, suggesting that regulation of bile salt synthesis is an evolutionary acquired trait specific for the vertebrate nuclear receptors. Two major evolutionary shifts in bile salt structure have been identified (50, 136) and hypothesized that the bile alcohols found in jawless fish (Agnatha) represents the “ancestral” bile salt phenotype from which the more “recent” vertebrate bile acids are derived (51). Secondary bile acids produced as a consequence of microbial metabolism in the gut are further detoxified through recognition by these nuclear receptors. In this regard, it is also notable that the related NRI family member, PXR that is well known for its xenobiotic detoxification role has also been shown to regulate intestinal inflammation by sensing bacterial metabolites (137). Furthermore, another unrelated xenobiotic receptor, the aryl hydrocarbon receptor, has been linked to the antibacterial response through sensing bacterial pigments and in enhancing IL-22 barrier immunity (138, 139).

While a comprehensive evolutionary history of LXR, FXR, and the other nuclear hormone receptors is beyond the present scope of this article; we believe the evolutionary path exemplified by VDR, in selecting dual functional roles in detoxification of harmful planar lipids and immune recognition, represents a central driver for the evolution of these receptors and evokes a general hypothesis for the coevolution of microbial–host metabolism underlying host-protection pathways.

## Author Contributions

HN, WD, and PG conceived, performed analysis, and wrote the paper.

## Conflict of Interest Statement

The authors declare that this document was written in the absence of any commercial or financial relationships that could be construed as a potential conflict of interest.

## Abbreviations

AluSx, *Alu* Sx subfamily; AHR, aryl hydrocarbon receptor; AMP, antimicrobial peptide; C_24_, 24-carbon; CAMP, cathelicidin antimicrobial peptide; CAR, constitutive androstane receptor; CBT, cytochrome *b* termination protein; CH25H, cholesterol 25-hydroxylase; CNS, central nervous system; CYP, cytochrome P450; DBD, DNA binding domain; DHR96, *Drosophila* hormone receptor-like in 96; EAE, experimental autoimmune encephalitis; EcR, ecdysone receptor; FXR, farnesoid X receptor; GKO, interferon-gamma knock out; GR, glucocorticoid receptor; HK, hexokinase; JAK/STAT, janus kinase/signal transducer and activator of transcription; IIS, insulin/insulin-like growth factor signaling; IL, interleukin; IFN, interferon; IRF, interferon response factor; ISG, interferon-stimulated gene; ISGF, interferon-stimulated gene factor; LBD, ligand-binding domain; LCA, lithocholic acid; LPS, lipopolysaccharide; LXR, liver X receptor; MAPK, mitogen-activated protein kinase; miR342-5p, microRNA 342-5p; MS, multiple sclerosis; NF-κB, nuclear factor-kappa B; NHR-8, nuclear hormone receptor family member nhr-8; NFAT, nuclear factor of activated T-cells; PPAR, peroxisome proliferator-activated receptor; PXR, pregnane X receptor; RAR, retinoic-acid receptor; ROR, RAR-related orphan receptor; RXR, retinoid X receptor; RSMAD, receptor-regulated SMAD; SMAD, mothers against decapentaplegic homolog; STING, stimulator of interferon genes; TGF-β, transforming growth factor-beta; Th2, type 2 T helper cell; TLR, toll-like receptor; TLR2/1L, toll-like receptor 2/1 ligand; USP, ultraspiracle; UVB, ultraviolet B; VDR, vitamin D receptor; VDRE, vitamin D response element; WGD, whole genome duplication.
